# Cross-Platform Comparison of Untargeted and Targeted Lipidomics Approaches on Aging Mouse Plasma

**DOI:** 10.1038/s41598-018-35807-4

**Published:** 2018-12-10

**Authors:** Kévin Contrepois, Salah Mahmoudi, Baljit K. Ubhi, Katharina Papsdorf, Daniel Hornburg, Anne Brunet, Michael Snyder

**Affiliations:** 1 Department of Genetics, Stanford University, 300 Pasteur Drive, Stanford, California, 94305 USA; 2 SCIEX, 1201 Radio Rd, Redwood City, California, 94065 USA

## Abstract

Lipidomics – the global assessment of lipids – can be performed using a variety of mass spectrometry (MS)-based approaches. However, choosing the optimal approach in terms of lipid coverage, robustness and throughput can be a challenging task. Here, we compare a novel targeted quantitative lipidomics platform known as the Lipidyzer to a conventional untargeted liquid chromatography (LC)-MS approach. We find that both platforms are efficient in profiling more than 300 lipids across 11 lipid classes in mouse plasma with precision and accuracy below 20% for most lipids. While the untargeted and targeted platforms detect similar numbers of lipids, the former identifies a broader range of lipid classes and can unambiguously identify all three fatty acids in triacylglycerols (TAG). Quantitative measurements from both approaches exhibit a median correlation coefficient (r) of 0.99 using a dilution series of deuterated internal standards and 0.71 using endogenous plasma lipids in the context of aging. Application of both platforms to plasma from aging mouse reveals similar changes in total lipid levels across all major lipid classes and in specific lipid species. Interestingly, TAG is the lipid class that exhibits the most changes with age, suggesting that TAG metabolism is particularly sensitive to the aging process in mice. Collectively, our data show that the Lipidyzer platform provides comprehensive profiling of the most prevalent lipids in plasma in a simple and automated manner.

## Introduction

Lipids play a key role in many biological processes, such as cellular structure, energy storage, and cell signaling, and are dysregulated in a variety of health conditions, including aging, cancer, diabetes, cardiovascular disorders and neurodegenerative diseases^1,2^. As such, there is a strong interest in the comprehensive analysis of lipids for biomarker discovery and for gaining novel insights into the onset and progression of diseases^3^. However, global profiling of the entire collection of lipids (known as lipidomics) is challenging because the lipidome comprises thousands of different molecular species that are highly diverse in chemical structure and composition^2^.

Mass spectrometry (MS) is the preferred technology to profile lipids as it provides resolution, sensitivity, selectivity, and throughput^4^. MS-based lipidomics can be performed using either untargeted or targeted approaches, each with their own set of advantages and limitations. Untargeted platforms are unbiased and have broad coverage, as they potentially detect all the lipids present in a sample^5,6^. However, they are often used in a semiquantitative manner (providing relative quantification) and certain aspects of the workflow, including data normalization and lipid identification, are challenging, time-consuming and not well standardized. Untargeted lipidomics can be done by direct infusion or liquid chromatography (LC)-based approaches^1^. LC-MS is often preferred because it offers higher sensitivity as well as more accurate lipid identification and quantification^7^. In contrast, targeted platforms traditionally focus on the absolute quantification of a small number of pre-defined lipids using isotopically labeled internal standards. The number of targets is often limited due to the lack of commercially available standards. Targeted approaches are high-throughput because data generation and analysis is fast and straightforward, quantitative, but usually have a more limited coverage.

Recently, a novel targeted lipid analysis technology has been developed called the Lipidyzer platform (SCIEX) that can quantify over 1,100 lipid molecular species across 10 lipid classes in human plasma/serum. The Lipidyzer platform estimates concentrations using a mixture of internal standards specifically designed for this purpose^8^. Consequently, the Lipidyzer platform may constitute a good compromise between untargeted and conventional targeted approaches, providing broad coverage, accurate quantification and fast and straightforward data processing. However, its performance has not been compared to more established untargeted lipidomics approaches and it has not been tested in a biological setting.

These questions prompted us to compare a conventional untargeted LC-MS approach with the targeted Lipidyzer platform. In this study, we first conducted a cross-platform comparison of the workflow, lipid coverage, precision and accuracy, and determined how well the two platforms correlate quantitatively with each other. We then applied both platforms to profile lipids in plasma from young and old mice, because a comprehensive description of changes in lipids during healthy aging in mice is still lacking. Altogether, our study constitutes the first cross-platform comparison of two state-of-the-art lipidomics platforms and provides the first assessment of the Lipidyzer platform in the context of aging.

## Results and Discussion

### Qualitative and quantitative cross-platform comparison

**Figure 1:**
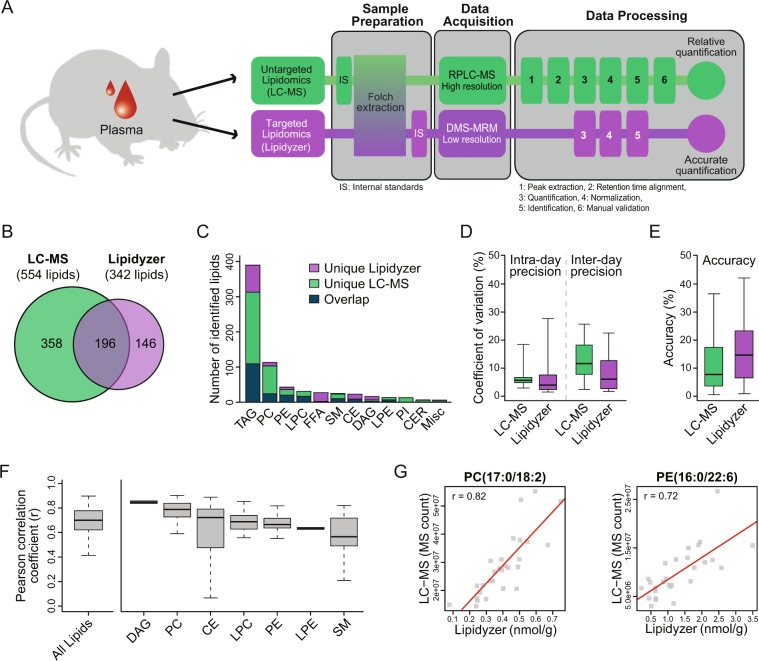
Qualitative and quantitative cross-platform comparison. (**A**) Schematic illustration of the workflows for both untargeted LC-MS and targeted Lipidyzer platforms. Lipids were extracted from mouse plasma using a modified Folch method. Two internal standards (IS) were spiked-in prior to lipid extraction and were used for normalization in the LC-MS approach. The Lipidyzer platform requires the addition of a mixture of IS that is used for quantification, which in this case was added to the lipid extracts prior to analysis. Processing of LC-MS data consisted in six steps: (1) peak extraction, (2) retention time alignment, (3) quantification, (4) normalization, (5) identification, and (6) manual validation. In contrast, the Lipidyzer platform quantifies a pre-determined list of lipids, a process that is automated by the software provided with the platform. While our untargeted LC-MS provides relative lipid abundances, the Lipidyzer platform gives an accurate quantification in nmol/g (estimated concentration) based on the IS. DMS: Differential Mobility Spectrometry, MRM: Multiple Reaction Monitoring. (**B**) Venn diagram representing lipid coverage of each approach and their overlap. Note that the lipids detected with the LC-MS approach were converted to match the level of information provided by the Lipidyzer platform. Lipid coverage assessment was performed using data from three injections of a pool sample. (**C**) Barplot depicting the overlap in coverage by lipid class. (**D**) Boxplot representing intra- and inter-day precisions with the indicated platform. Precisions were calculated on IS spiked in a plasma matrix using five replicate injections on the same day (intra-day) and on three consecutive days (inter-day). Lipidomics Workflow Manager (LWM) recommended concentrations spanning 3 orders of magnitude were used to determine precisions. (**E**) Boxplot depicting accuracies of both platforms using IS at LWM recommended concentrations. Accuracy was calculated on each IS using a 7-point dilution series. (**F**) Pearson correlation coefficient of lipids identified by both platforms across all biological samples (87 lipids). TAG were excluded from the analysis because of nomenclature discrepancies between the two approaches. (**G**) Scatterplots showing examples of one PC and one PE with a Pearson correlation coefficient close to the median of the corresponding lipid class. TAG: triacylglycerol, DAG: diacylglycerol, PC: phosphatidylcholine, PE: phosphatidylethanolamine, PI: phosphatidylinositol, LPC: lysophosphatidylcholine, LPE: lysophoshatidylethanolamine, SM: sphingomyelin, CER: ceramide, CE: cholesterol ester, FFA: free fatty acid, MISC: miscellaneous.

We performed a comprehensive comparison between an established untargeted LC-MS approach and the targeted Lipidyzer platform, by comparing the workflows associated with each respective platform, and assessing their ability to detect and quantify lipids in mouse plasma. A key difference between the two platforms resides in lipid data acquisition, which is performed in fundamentally different ways (Fig. 1A). The untargeted LC-MS approach acquires data by first separating lipid species by reverse phase liquid chromatography (RPLC) and then detecting the molecular ions by high resolution mass spectrometry. In contrast, the targeted Lipidyzer platform separates lipid classes by differential mobility spectrometry (DMS)^13^ and then detects lipid species by multiple reaction monitoring (MRM) using low resolution mass spectrometry. Since the Lipidyzer quantifies a pre-determined list of lipids, data processing is faster and more straightforward (*i.e*. elimination of the lipid identification step). Lipid abundance are reported as estimated concentrations using a mixture of internal standards (IS) designed to correct for diversity in fatty acid chain length and degree of unsaturation^8^ (see Materials and Methods for details). These steps are automated by the software provided with the platform, which ultimately reports a data matrix with calculated concentrations in nmol/g. In contrast, our untargeted LC-MS approach provides relative abundances of all the lipids detected in a sample. Although using the same mixture of internal standards would allow for a similar estimation of lipid concentration as the Lipidyzer, the data processing - including data normalization and lipid identification - is challenging and more time-consuming. For instance, it is still necessary to manually validate lipid identities because lipid complexity may lead to co-elution and/or incorrect assignments (Materials and Methods).

We next assessed lipid coverage for each approach and determined the overlap of identified lipids. Both methods had a broad coverage with a total number of 337 and 342 lipids detected across 11 lipid classes with the LC-MS and the Lipidyzer platforms, respectively (see Supplementary Table 1A,B for a list of identified lipids). These numbers were similar to what is commonly detected in lipidomics laboratories using human plasma^18^. Although both platforms provide the same level of information for most lipid species, there are discrepancies in the nomenclature of triacylglycerols (TAG) and sphingomyelins (SM). While the untargeted LC-MS platform identifies all three fatty acids (FA) that compose individual TAG (*e.g*. TAG(16:0/18:1/18:2)), the targeted platform identifies one fatty acid and provides the total number of carbons and degree of unsaturation (*e.g*. TAG52:3-FA16:0). This annotation is useful for fatty acid composition analysis but overestimates the number of TAG reported. Moreover, while the untargeted LC-MS approach provides the total number of carbons and degree of unsaturation in SM (*e.g*. SM(d38:1)), the targeted approach assumes an 18:1 sphinganine backbone and reports the other FA (*e.g*. SM(20:0)). Hence, LC-MS is advantageous over Lipidyzer in accurately identifying individual TAG species. In order to determine the overlap between the identified lipids across the two platforms, the lipids detected with the LC-MS approach were converted to match the level of information provided by the Lipidyzer platform (Materials and Methods). The conversion extended the number of identified lipid molecular species by LC-MS to 554 (compared to 337 before conversion). A comparison of both platforms resulted in 196 overlapping lipid species (35% and 57% of the lipids detected with the untargeted and targeted approaches, respectively) (Fig. 1B). This result is comparable to the overlap described in the NIST study^18^ where the authors found 226 lipids in common with the LIPIDMAPS study^19^ which represents 67% and 38% of the detected lipids, respectively. Across both platforms, most identified lipids were TAG and phosphatidylcholines (PC) (Fig. 1C), which reflects their higher abundance in blood plasma^19^. Many lipids were only detected with one platform. While the targeted approach uniquely detected free fatty acids (FFA) and many cholesterol esters (CE), the untargeted LC-MS approach detected many PC, particularly ether-linked PC (*i.e*. plasmalogens) and phosphatidylinositols (PI). Thus, these approaches are also complementary to each other and when used together increase the lipid coverage, reaching a total of 700 lipid molecular species detected in mouse plasma. It is important to note that despite the fact that the main lipid classes (in abundance) were covered by both approaches^17,18^, other lipid classes including acylcarnitines, oxylipins, fatty acid esters of hydroxyl fatty acids (FAHFA), bile acids, phospatidylserines (PS), phosphatidylglycerols (PG), and phosphatidic acids (PA) were not included in the list of lipids targeted by the Lipidyzer platform. These lipids were also not detected by the LC-MS approach either due to low abundance and/or LC method that is not suited to retain certain classes.

We then calculated the intra- and inter-day precisions using 54 deuterated IS covering 10 lipid classes that were spiked in a plasma matrix at physiological concentrations across 3 orders of magnitude. We observed a median coefficient of variation (CV) of 3.1% and 4.7% for intra-day precision and a median CV of 10.6% and 5.0% for inter-day precision with the LC-MS and Lipidyzer platforms, respectively (Fig. 1D). We also determined the technical repeatability across all identified lipids using a triplicate injection of the same biological sample (*i.e*. pool sample). Technical repeatability was high for both platforms, with a median CV of 6.9% and 4.7% for the untargeted and targeted approaches, respectively (Supplementary Figure 1A). These values are comparable or even exceed the median CV recently reported for 9 LC-MS platforms (5.5–10%)^17^. In addition, we calculated a median accuracy of 6.9% and 13.0% with the LC-MS and Lipidyzer platforms, respectively (Fig. 1E). The LC-MS approach exhibited slightly better accuracy due to the fact that the signal measured on the Lipidyzer had a tendency to plateau at high concentrations for certain classes – namely TAG, DAG, CE and CER (Supplementary Figures 2 and 3). Indeed, the accuracy of the Lipidyzer was improved when discarding the highest concentration sample from the calibration curves, yielding accuracies comparable to the LC-MS approach (Supplementary Figure 1B).

Finally, we assessed how well the quantitative measurements obtained by the two platforms correlated with each other. To this end, we first calculated the Pearson correlation coefficient (r) for the lipids that were detected by both platforms. We observed a median correlation of 0.71 across all identified lipids (Fig. 1F), with a higher correlation in lipids that exhibited a larger biological variability across the samples (Supplementary Figure 1C). Similar correlations were observed within each lipid class (Fig. 1F,G). This correlation is greater than what has been reported for metabolites, comparing untargeted LC-MS and targeted approaches (0.44–0.69)^20–22^. In order to assess inter-platform correlation independently of biological variability, we compared lipid intensities using dilution series of IS spanning more than 2 orders of magnitude spiked in a plasma matrix. Correlation coefficients were high with a median r of 0.99 (Supplementary Figure 4). These results show that quantitative data from the untargeted LC-MS approach are well correlated to data from the targeted Lipidyzer platform.

Collectively we found that both untargeted and targeted lipidomics platforms were efficient in profiling numerous diverse lipids in mouse plasma with similar precision and accuracy. Importantly, both approaches were well correlated across all the detected lipid classes. The Lipidyzer platform offers the advantages of providing accurate quantification of a broad range of lipids species across many classes in a fast and streamlined fashion. In contrast, the LC-MS approach is unbiased and has therefore the potential to discover new and/or modified lipids. Also, the separation by liquid chromatography provides more accurate lipid identification and higher quantification accuracy for certain lipid classes, such as TAG by avoiding isobar and isomer interferences.

### Comparative analysis of changes in mouse plasma lipids with age

**Figure 2:**
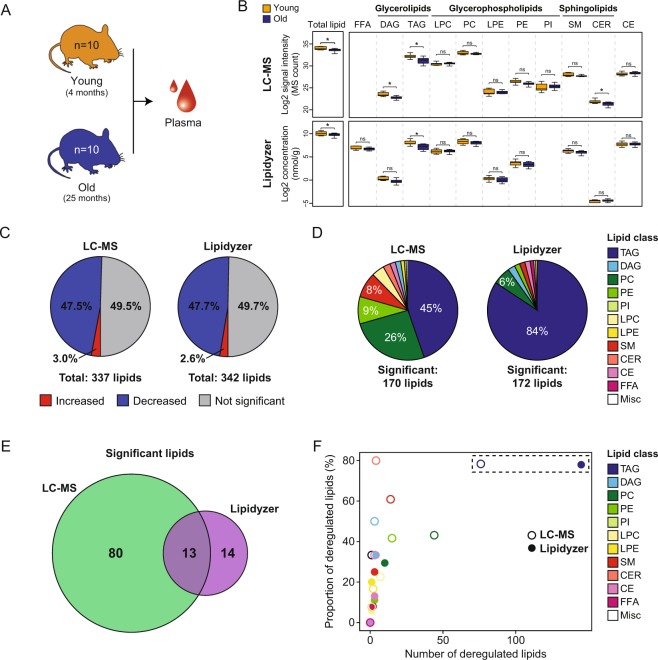
Comparative analysis of changes in mouse plasma lipids with age. (**A**) Schematic illustration of sample collection. (**B**) Boxplot showing the distribution of total plasma lipid content and different lipid classes in young and old mice. *P < 0.05, ns: Not Significant. (**C**) Pie chart depicting the proportion of detected lipids that are significantly increased (red), decreased (blue) or do not change (grey) with age (FDR < 0.05). (**D**) Pie chart showing the proportion of different classes that change with age. (**E**) Venn diagram representing the overlap of deregulated lipids detected with the LC-MS and Lipidyzer platforms. TAG were excluded from this analysis because of nomenclature discrepancies between the two approaches. (**F**) Proportion and number of deregulated lipids within each class. TAG: triacylglycerol, DAG: diacylglycerol, PC: phosphatidylcholine, PE: phosphatidylethanolamine, PI: phosphatidylinositol, LPC: lysophosphatidylcholine, LPE: lysophoshatidylethanolamine, SM: sphingomyelin, CER: ceramide, CE: cholesterol ester, FFA: free fatty acid, MISC: miscellaneous.

We next tested the ability of the two platforms to detect physiological differences in lipid abundance between biologically distinct groups by comparing the lipid profiles of plasma from young (4 months, hereinafter referred to as young plasma, n = 10) and old (25 months, hereinafter referred to as old plasma, n = 10) mice (Fig. 2A). Both platforms detected modest global decline in total plasma lipid levels with age (*P* = 0.01 with LC-MS, *P* = 0.07 with Lipidyzer) (Fig. 2B). The global decrease was primarily due to reduced levels of TAG (*P* = 0.02 with LC-MS, *P* = 0.03 with Lipidyzer). We also observed a decrease in diacylglycerols (DAG, *P* = 0.02) and ceramides (CER, *P* = 0.03) with the untargeted approach, but this was not observed with the targeted platform (*P* = 0.13 for DAG, *P* = 0.27 for CER). This difference was likely due to specific lipid coverage of each method. We next examined differences in specific lipid species between young and old plasma. Differential analysis revealed that half of the detected lipids (170 and 172 lipids with the LC-MS and Lipidyzer platforms, respectively) were significantly different between young and old plasma (FDR < 0.05) (Fig. 2C and Supplementary Table 1A,B). Consistent with a decline in total lipid level with age, most lipid species were decreased, and only a small portion (2–3%) were increased. A cross-platform comparison of the deregulated lipids with age revealed that the untargeted platform detected a more diverse set of lipids than the targeted platform (Fig. 2D). Both platforms primarily detected changes in TAG that represent 45% and 84% of the significantly deregulated lipids for LC-MS and Lipidyzer, respectively. A greater proportion of the lipids detected by LC-MS belonged to other classes including PC (26% vs 6% with Lipidyzer), PE (9% vs 2% with Lipidyzer) and SM (8% vs 2% with Lipidyzer). Among the deregulated lipids (excluding TAG), 13 lipids were detected with both platforms and changed in the same direction (Fig. 2E and Supplementary Table 1A,B). The remaining lipids were detected with only one platform (80 and 14 identified with untargeted and targeted approaches, respectively), reflecting the unique lipid coverage of each platform. Interestingly, TAG was the most susceptible lipid class to aging, with 78% of the individual TAG species deregulated across both platforms (Fig. 2F). In line with a global decline of plasma TAG levels, near all individual TAG decreased with age. This is consistent with what was previously reported using a standard colorimetric clinical test in mice^23^. However, our study goes beyond the previous findings by providing a list of individual TAG that change with age. TAG are the main lipid components of dietary fat, are stored in adipose tissue and are used as an energy source for the organism^24^. Since the mice were fasted 12 h prior to sacrifice, these differences are not likely due to food intake. Instead, they could be indicative of an enhanced TAG deposition or degradation in tissues, or a decreased TAG biosynthesis.

Collectively, these results demonstrate that both platforms are efficient in robustly detecting changes in mouse plasma lipids with age at the class and molecular species levels. While the untargeted LC-MS approach detected a more diverse set of lipids deregulated with age, comparable data could be collected and analyzed in a fast and streamlined fashion with the Lipidyzer platform. Though previous studies have started to look at alterations in plasma lipids with age, they have mainly provided information on free fatty acids and/or total lipid class levels^23,25–27^. In this study, we go beyond these previous observations by assessing the levels of 700 individual lipids with age. Thus, our study constitutes one of the most comprehensive lipid profiling of aging plasma to-date, and identifies TAG as the most susceptible lipid class to aging in mice.

## Conclusion

Our cross-platform comparison reveals that both untargeted and targeted platforms efficiently and robustly profile lipids from mouse plasma and that the quantitative measurements acquired with both platforms correlate well. The untargeted LC-MS approach is the optimal choice for identifying novel or modified lipids and lipid classes not covered by the Lipidyzer (*i.e*. PI, ether-linked PC). Due to its broader coverage, it also identified a more diverse set of dysregulated lipids in aging mouse plasma. In addition, untargeted LC-MS is versatile and can be applied to other biological materials such as liver, skeletal muscle and adipose tissue. However, this approach requires a higher level of application expertise, and data processing can be time-consuming. In contrast, the Lipidyzer platform detects and quantifies a wide variety of plasma lipids in an efficient manner by overcoming the data processing challenges. Presently, the mixture of internal standards used to accurately quantify lipids has only been optimized and validated for human plasma and serum, and thus Lipidyzer in its current form is probably not ideal for profiling other tissue types with very different lipid profiles. Furthermore, the continuous use of internal standards can be costly. Collectively, our data show that the Lipidyzer platform constitutes a good compromise between untargeted and conventional targeted approaches, providing a comprehensive profiling of the most prevalent lipids in plasma in a simple and automated manner.

## Materials and Methods

### Chemicals

LC-MS-grade solvents and mobile phase modifiers were obtained from Fisher Scientific (water, acetonitrile, methanol, isopropanol, formic acid), Sigma–Aldrich (ammonium acetate, MTBE) and Acros Organics (chloroform).

### Animals

All animals were treated and housed in accordance with the Guide for Care and Use of Laboratory Animals. All experimental procedures were approved by Stanford’s Administrative Panel on Laboratory Animal Care (APLAC) and were in accordance with institutional and national guidelines. Male C57BL/6 mice at different ages (4 and 25 months) were obtained from the National Institute on Aging (NIA) colony, and were acclimated in the animal facility at Stanford University for 1 month before being processed. No live animals were censored.

### Plasma preparation

Mice were sacrificed after overnight fasting (~12 h) and blood was immediately collected by cardiac puncture into a tube containing EDTA (10% v/v, 0.5 M EDTA). Plasma was prepared by two consecutive centrifugation steps at 500 rcf and 13,000 rcf, respectively, each for 10 minutes at room temperature, and then aliquoted and stored at −80 °C before further analysis.

### Lipid extraction

Lipids were extracted from plasma using a biphasic separation with cold chloroform, methanol and water^9^. Briefly, 600 μl of 2:1 chloroform:methanol mixture was added to 30 μl of plasma, vortexed for 1 min at room temperature and incubated under agitation for 1 h at 4 °C. The solvent mixture contained two internal standards (IS) from Avanti Polar Lipids (PC(17:0/14:1(9Z)), cat#: lm-1004 and TAG d5-(17:0/17:1(10Z)/17:0), cat#: 110544) to control for extraction efficiency, evaluate LC-MS performance and normalize the LC-MS data. After addition of 100 μl of 0.9% sodium chloride (NaCl) in water, the samples were vortexed for 1 min and centrifuged at XXX g for 10 min at 4 °C. The lower phase containing the lipids was then collected and dried down under nitrogen. The dry extracts were reconstituted with 100 μl of 2:1 chloroform:methanol.

### Untargeted lipidomics by LC-MS

Lipid extracts were diluted 6-fold with 1:1:1 isopropanol/acetonitrile/H2O and analyzed using an Ultimate 3000 RSLC system coupled with a Q Exactive plus mass spectrometer (Thermo Scientific). Each sample was run twice in positive and negative ionization modes for a total run time of 62 minutes per sample. It should be noted that the total run time for RPLC-MS lipidomics experiments can be reduced by using polarity switching^10^, columns with sub-2 μm totally porous particles or particles with porous shell fused to a solid core^11^. 2 μl of plasma equivalent was used in total for the analysis. Data quality was ensured by (i) sample randomization for lipid extraction and data acquisition, (ii) 6 injections of a pool sample (*i.e*. equimolar mix of all the samples in the study) to equilibrate the LC-MS system prior to run the sequence, (iii) injection of a pool sample every 7 injections to control for signal deviation with time, (iv) discarding features from solvent blanks, (v) checking mass accuracy, retention time and peak shape of IS in every samples.

#### LC conditions

Lipids were separated using an Accucore C18 column 2.1 × 150 mm, 2.6 μm (Thermo Scientific) and mobile phase solvents consisted in 10 mM ammonium acetate and 0.1% formic acid in 60/40 acetonitrile/water (A) and 10 mM ammonium acetate and 0.1% formic acid in 90/10 isopropanol/acetonitrile (B). The gradient profile used was 30% B for 3 min, 30–43% B in 2 min, 43–55% B in 0.1 min, 55–65% B in 10 min, 65–85% B in 6 min, 85–100% B in 2 min and 100% B for 5 min. Lipids were eluted from the column at 0.4 ml/min, the oven temperature was set at 45 °C, and the injection volume was 5 μl. Autosampler temperature was set at 20 °C to prevent lipid aggregation.

#### MS conditions

The Q Exactive plus was equipped with a HESI-II probe and operated in full MS scan mode for all the samples. MS/MS spectra were acquired in data-dependent acquisition mode on pool samples. The source conditions were as follows: Spray Voltage = 3.5 kV (both ESI pos. and neg.), Vaporizer = 200 °C, Capillary Temp. = 375 °C, S-Lens = 55.0%, Sheath Gas = 40, Auxiliary gas = 8, Sweep Gas = 0. The acquisition settings were as follows: AGC (MS) = 3e6, AGC (MS2) = 1e5, Maximum Injection Time (MS) = 200 ms, Maximum Injection Time (MS2) = 50 ms, Mass Range = 100–1,500 Da, Resolution MS = 70,000 (FWHM at m/z 200), Resolution MS2 = 35,000 (FWHM at m/z 200), Top-10 experiments, Isolation Window = 1.0 m/z, Dynamic Exclusion = 12 s, Normalized Collision Energy (NCE) = 25–30. To maximize the number of identified lipids, the 100 most abundant peaks found in blanks were excluded from MS/MS events. External calibration was performed using an infusion of Pierce LTQ Velos ESI Positive Ion Calibration Solution or Pierce ESI Negative Ion Calibration Solution.

#### Data processing

LC-MS data were analyzed using an in-house analysis pipeline written in R (version 3.0.1). Lipid features (characterized by a unique mass/charge ratio and retention time) were extracted, aligned and quantified with the “XCMS” package (version 1.39.4) after conversion of .RAW files to .mzXML using the ProteoWizard MS convert tool. Grouping and annotation were performed with the “CAMERA” package (version 1.16.0). Features from blanks were discarded and data were normalized using the mean of the ratio of the two spiked IS. Only lipids present in >50% of the samples were kept for further analysis and missing values were imputed by the minimum value. When lipids were detected in multiple adduct forms, the most abundant ion was used for quantification. Lipids were identified by matching the precursor ion mass to a database and the experimental MS/MS spectra to a spectral library containing theoretical fragmentation spectra using LipidSearch software version 4.1 (Thermo Scientific)^12^. In order to reduce the risk of misidentification, each identified lipid was manually investigated to validate the assignments. The manual validation consisted in verifying that 1) both positive and negative mode MS/MS spectra match the expected fragments, 2) the main lipid adduct forms detected in positive and negative modes are in agreement with the lipid class identified, 3) the retention time is compatible with the lipid class identified and 4) the peak shape is acceptable. The fragmentation pattern of each lipid class detected in mouse plasma was experimentally validated using the SPLASH Lipidomix (Avanti Polar Lipids, cat#: 330707).

### Targeted lipidomics using the Lipidyzer platform

The Lipidyzer platform comprises a 5500 QTRAP System equipped with a SelexION differential mobility spectrometry (DMS) interface (SCIEX) and a high flow LC-30AD solvent delivery unit (Shimazdu). The DMS has proven very successful in fractionating lipid classes in gas phase^13^ which reduces isobaric interference inherent in lipid analysis and yields simplified and more reliable data for qualitative and quantitative analysis. In addition, DMS eliminates the need for any up-front chromatographic separation which greatly simplifies the workflow and increases the throughput. Each sample is run once on the platform using a method that combines DMS ‘on’ and ‘off’ as well as positive and negative ionization modes. The following lipid classes were quantified with i) DMS ‘on’ and in negative ionization mode: PC, PE, LPC, LPE, ii) DMS ‘on’ and in positive ionization mode: SM, iii) DMS ‘off’ and in negative ionization mode: FFA, iv) DMS ‘off’ and in positive ionization mode: TAG, DAG, CE, CER. Data acquisition lasted for 21 minutes per sample and 20 μl of plasma equivalent was injected. Data quality was ensured by (i) tuning the DMS compensation voltages using a set of lipid standards (SCIEX, cat#: 5040141) after each cleaning, more than 24 hours of idling or 3 days of consecutive use, (ii) performing a quick system suitability test (QSST) (SCIEX, cat#: 50407) before each batch to ensure acceptable limit of detection for each lipid class, (iii) sample randomization for lipid extraction and data acquisition, and (iv) triplicate injection of lipids extracted from a reference plasma sample (SCIEX, cat#: 4386703) at the beginning of the batch.

#### LC Conditions

Flow injection analysis (FIA) was performed at 7 μl/min using a running solution that consisted in 10 mM ammonium acetate in 50/50 dichloromethane:methanol. Minimal carryover (<0.5%) was achieved by using PEEKsil tubing and washing at 30 μl/min for two minutes between injections. The chemical modifier 1-propanol was added to the curtain gas (transport gas for the DMS cell) in order to improve DMS separation as previously described^13^.

#### DMS conditions

The DMS is a small planar cell located at atmospheric pressure in front of the mass spectrometer orifice between the ESI source and the high vacuum region of the instrument. An RF (radio frequency) voltage is applied across the cell that is cycled between high and low fields. As the ions transit through the cell, they are separated based on the difference in their mobility. A second voltage (compensation voltage, COV) is applied to steer analytes through the cell into the mass spectrometer^14^. DMS lipid class separation works on the principle that each lipid class has a different head group dipole moment. Hence, by applying a specific COV, each lipid class can be directed through the mobility cell and into the MS while all other classes are not transmitted. By ramping the COV, different classes of lipids transit successfully through the cell, thereby allowing sequential analysis of all lipid classes one at a time. The transmission of ions through the DMS lasts 25 ms at which point ions enter the MS for multiple reaction monitoring (MRM) analysis. DMS parameter settings were set as follows: Temperature = Low, Separation Voltage = 3.5 kV and DMS resolution = Low.

#### MS conditions

Lipid molecular species were quantified by a 5500 QTRAP mass spectrometer in MRM and positive/negative ionization switching. Lipid classes were quantified using the following ion forms: [M + NH4]+ for CE, DAG and TAG, [M + H] + for SM and CER, [M − H]- for LPE, PE and FFA, and [M + CH3COO]- (acetate adduct) for LPC and PC. These ion forms were selected since they are the most abundant ions in the ionization mode that provides fatty acid composition upon fragmentation. Source and gas setting were as follow: Curtain Gas = 17, CAD Gas = Medium, Ion Spray Voltage = 4.1 kV in positive mode and −2.5 kV in negative mode, Temperature = 200 °C, Nebulizing Gas = 17 and Heater Gas = 25. Twenty spectral scans were collected for each lipid of which 4 outlier scans were discarded and 16 remaining scans were averaged and used to estimate the concentration of each detected lipid (see below).

#### Data processing

Lipidyzer data were analyzed using the Lipidomics Workflow Manager (LWM) software written specifically for this platform. The software allows for automated data acquisition, processing and reporting. Accurate quantification is performed using 54 deuterated IS developed with Avanti Polar Lipids that cover the 10 main lipid classes (in abundance) found in plasma/serum^15^ (SCIEX, cat#: 5040156, lot#: LPISTDKIT-101) (see Supplementary Table 2 for a complete list). The diversity of fatty acid chain lengths and degrees of unsaturation result in differential fragmentation efficiency which impacts quantitation^16^. To minimize this phenomena, most lipid classes have multiple IS with varied fatty acid composition. Each MRM Q1/Q3 pair is specific to a lipid species where Q1 is the precursor mass and Q3 is the mass of one of its constituent fatty acid product ions. For a particular lipid species the software calculates concentration as average intensity of the analyte MRM/average intensity of the most structurally similar IS MRM multiplied by its concentration in nmol/ml. Lipid concentration is then converted in nmol/g with the assumption that 1 ml of plasma is equal to 1 g. Quantitative results were validated at the class level against traditional GC-MS-based methodologies.

### Quantification precision and accuracy

Precision and accuracy were calculated on the mix of 54 deuterated standards designed for the Lipidyzer platform (SCIEX, cat#: 5040156, LPISTDKIT-101), which were spiked in lipid extracts from a reference human plasma sample (SCIEX, cat#: 4386703). Internal standards covered 10 main lipid classes found in human plasma with different final concentrations reflecting their physiological concentrations. LWM recommended concentrations spanning 3 orders of magnitude were used to determine precisions (Supplementary Table 2). Lipid extracts were prepared using methanol, methyl tert-butyl ether (MTBE) and water as previously described^17^ and stored at −20 °C in 10 mM ammonium acetate in 90/10 methanol/toluene before analysis. Intra-day precision was calculated as coefficient of variation (CV) using five replicate injections on the same day. Inter-day variability was determined by calculating the CV between the median of five replicate injections across three consecutive days. Precisions were calculated after median normalization for LC-MS data. Calibration curves were constructed for each IS using a 7-point dilution series spanning more than 2 orders of magnitude around the LWM recommended spike-in concentration (see Supplementary Table 2 for details). The determination coefficient (r^2^) was calculated for each IS calibration curve (Supplementary Figures 2 and 3). Accuracy was determined for each IS at the LWM recommended concentration by calculating the percentage of deviation of the calculated concentration relative to the actual concentration as follows:

$$ \% \,accuracy=|(calculated\,concentration-actual\,concentration)\,\div\,actual\,concentration|\times 100$$

### Conversion of lipid identities

There were discrepancies in the level of information provided for triacylglycerols (TAG) and sphingomyelins (SM) between the two platforms. In order to calculate the overlap, lipids identified by LC-MS were converted to match the level of information provided by the Lipidyzer. LC-MS identifies all three fatty acids (FA) contained in TAG (*e.g*. TAG(16:0/18:1/18:2)) however, Lipidyzer identifies one fatty acid and provides the total number of carbons and unsaturations (*e.g*. TAG52:3-FA16:0). In the case of TAG(16:0/18:1/18:2), it was converted to TAG52:3-FA16:0, TAG52:3-FA18:1, and TAG52:3-FA18:2. For SM, LC-MS provides the total number of carbons and unsaturations (*e.g*. SM(d38:1)). Fatty acid composition is difficult to obtain because SM poorly fragment under the conditions used. Lipidyzer assumes an 18:1 sphinganine backbone and reports the other FA chain (*e.g*. SM(20:0)). Since SM containing 18:1 sphinganine backbone are the most prevalent forms in plasma, SM(d38:1) was converted to SM(20:0). Ultimately, the LC-MS approach detected 337 lipids that were converted to 554 lipid annotations to enable direct comparisons to the Lipidyzer platform.

### Statistical analysis and data visualization

No statistical methods were used to predetermine sample size. A two-tailed parametric Welch’s T-test was used for statistical comparison between young and old plasma lipids. *P*-values were corrected for multiple hypothesis testing using q-value correction. A FDR of 0.05 or less was considered significant. R v.3.2.0 was used for all statistical analysis. The boxplots depict the median and interquartile range, with whiskers indicating minimum and maximum values.

## Electronic supplementary material

Supporting Information

Supplementary Figures

Supplementary Dataset 1

Supplementary Dataset 2

Supplementary Dataset 3

## Data Availability

The datasets generated during and/or analyzed during the current study are available from the corresponding author on reasonable request.
