# Supplementary material for: Cross-Platform Comparison of Untargeted and Targeted Lipidomics Approaches on Aging Mouse Plasma
Source: Sci Rep. 2018 Dec 10;8:17747. doi: 10.1038/s41598-018-35807-4 (PMC6288111; doi:10.1038/s41598-018-35807-4)
Supplement: Supplementary file 1 — Supporting Information [file 41598_2018_35807_MOESM1_ESM.docx]

**Supporting Information**

**Cross-Platform Comparison of Untargeted and Targeted Lipidomics Approaches on Aging Mouse Plasma**

Kévin Contrepois^1,#^, Salah Mahmoudi^1,#^, Baljit K. Ubhi^2^, Katharina Papsdorf^1^, Daniel Hornburg^1^, Anne Brunet^1^, Michael Snyder^1,*^

^1^Department of Genetics, Stanford University, 300 Pasteur Drive, Stanford, California 94305, USA.

^2^ SCIEX, 1201 Radio Rd, Redwood City, California 94065, USA.

^*^Correspondence: mpsnyder@stanford.edu

^#^These authors contributed equally to this work

**Table of contents**

- **Supplementary figures (Figures S1 to S4)**
- **Supplementary tables (Tables S1 to S3)**

**Table S1. Differential analysis of young and old mouse plasma using the untargeted LC-MS and targeted Lipidyzer platforms.**

**Table S2. Internal standard concentrations used to generate calibration curves.**

**Table S3. Calibration curve coefficients of determination (r^2^)**

**SUPPLEMENTARY FIGURE LEGENDS**

**Figure S1.** A) Technical repeatability based on a triplicate injection of the same sample (*i.e.* pool sample) using all the lipids detected with the indicated platform. B) Boxplot depicting accuracies for each IS at concentrations recommended by Lipidomics Workflow Manager (LWM) on both platforms. Accuracy was calculated using 6-point dilution series with the higher concentration sample discarded (related to **Fig. 1E**). C) Scatterplots showing the correlation between Pearson correlation coefficients and biological variability with both approaches (related to **Fig. 1F**).

**Figure S2. Calibration curves obtained with the untargeted LC-MS platform.** Correlation is indicated as determination coefficient (r^2^).

**Figure S3. Calibration curves obtained with the targeted Lipidyzer platform.** Correlation is indicated as determination coefficient (r^2^).

**Figure S4. Correlations between LC-MS and Lipidyzer platforms using a 7-point dilution series**. Correlation is indicated as correlation coefficient (r).
