# Supplementary figures and images for: Cross-Platform Comparison of Untargeted and Targeted Lipidomics Approaches on Aging Mouse Plasma
Source: Sci Rep. 2018 Dec 10;8:17747. doi: 10.1038/s41598-018-35807-4 (PMC6288111; doi:10.1038/s41598-018-35807-4)

Figure S1

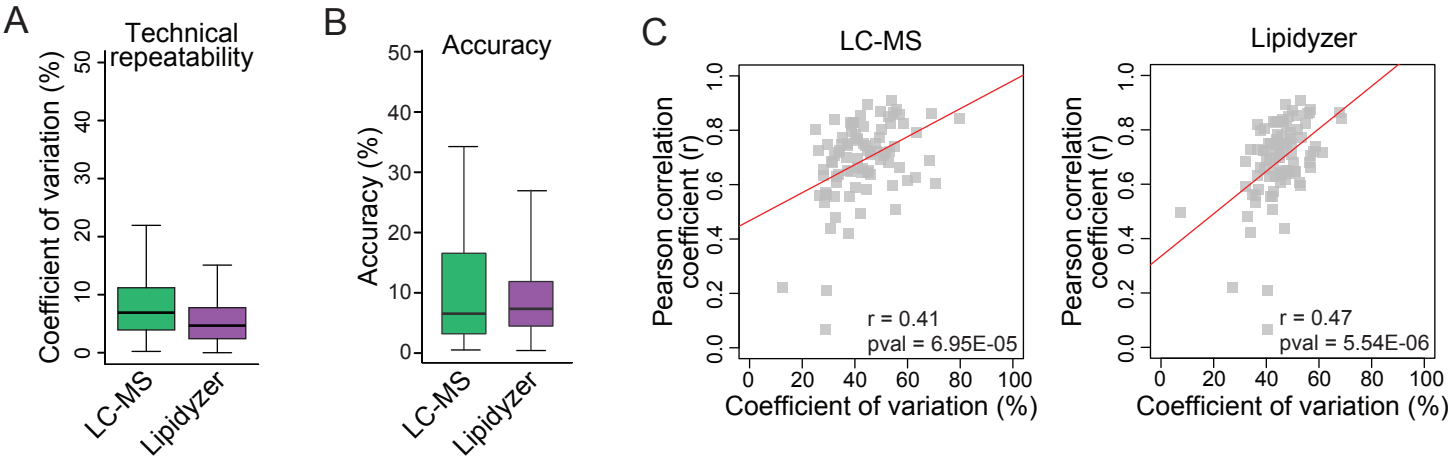

Figure S2 LC-MS platform

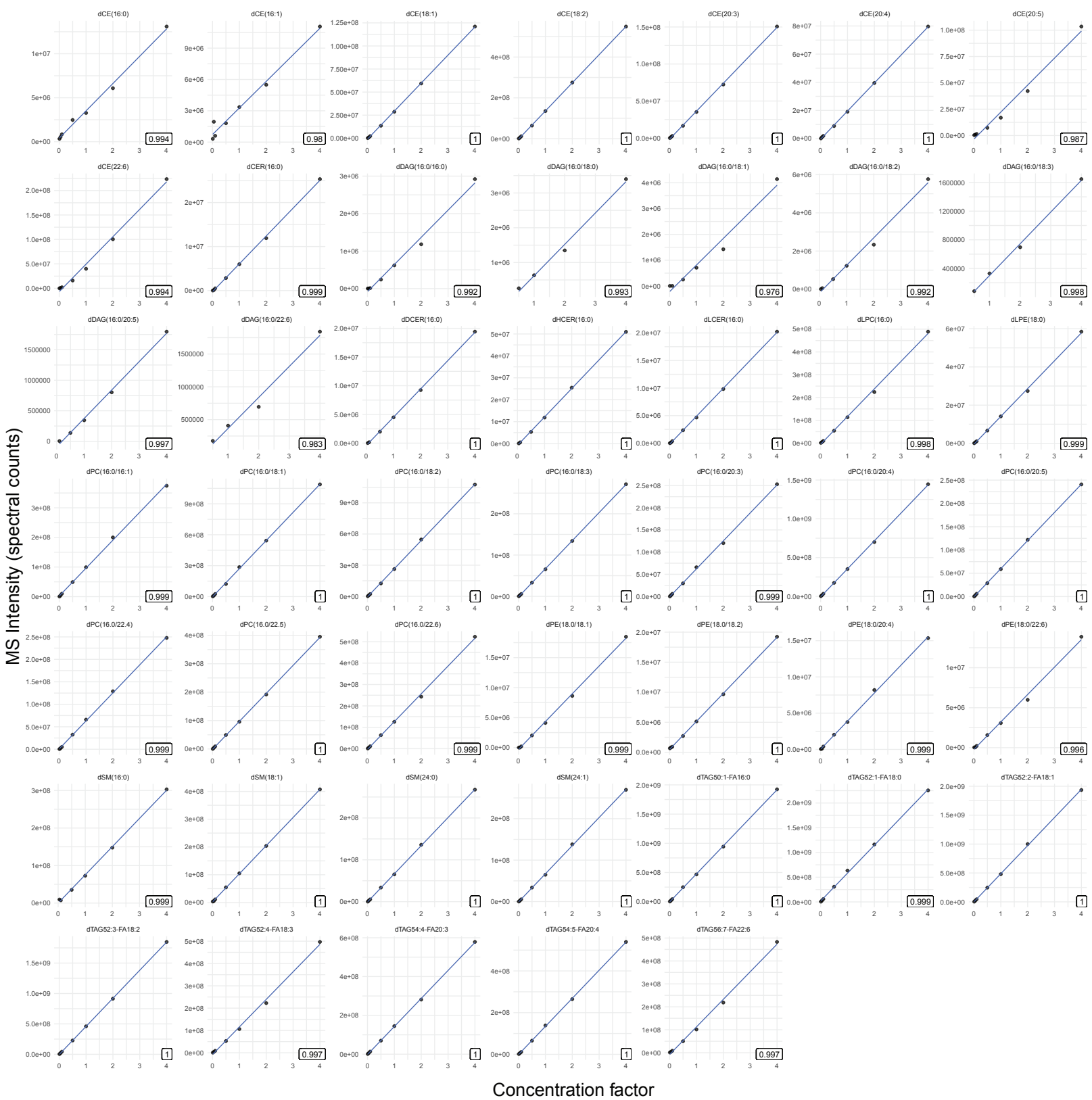

Figure S3 Lipidzyzer platform

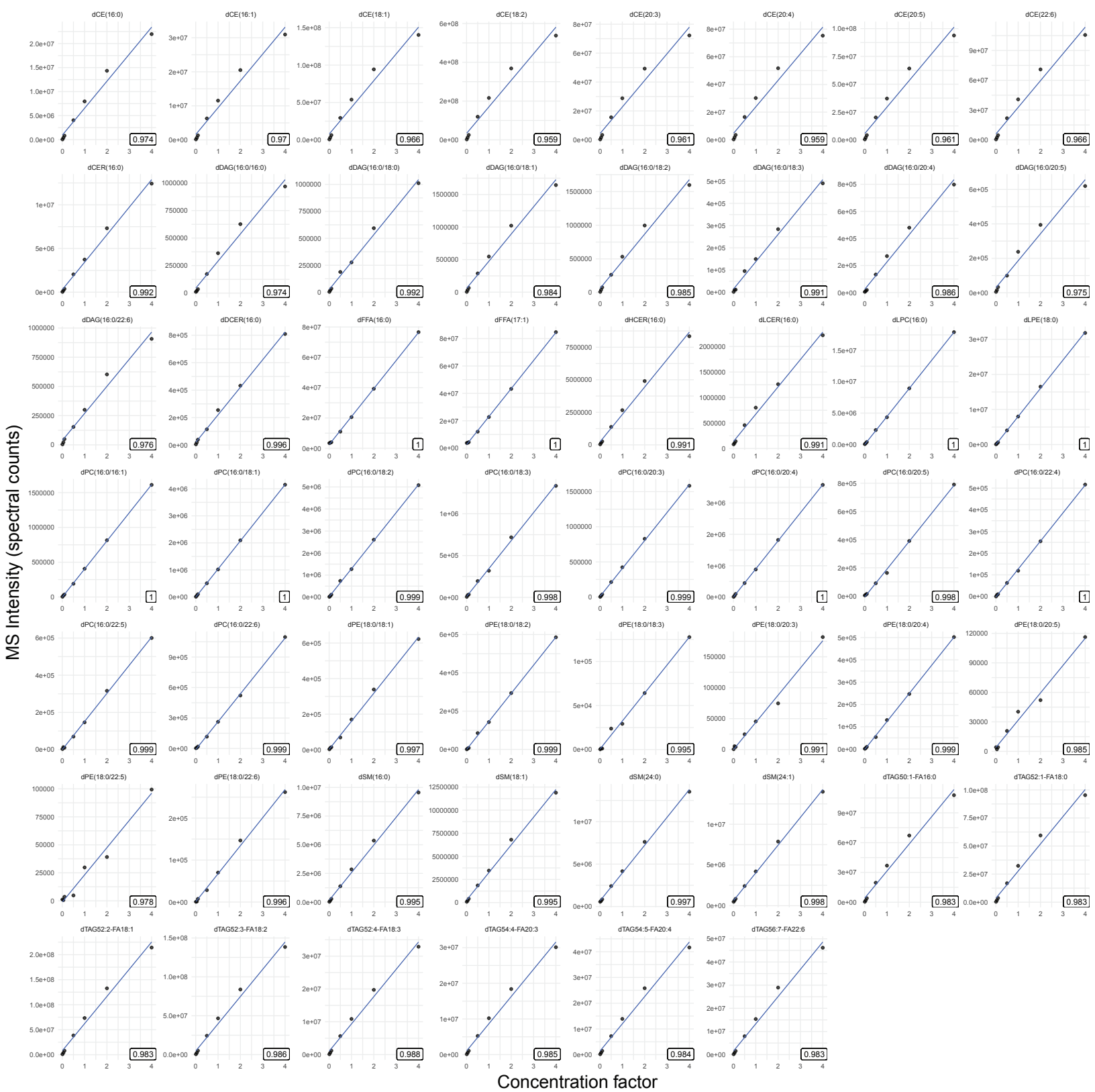

Figure S4

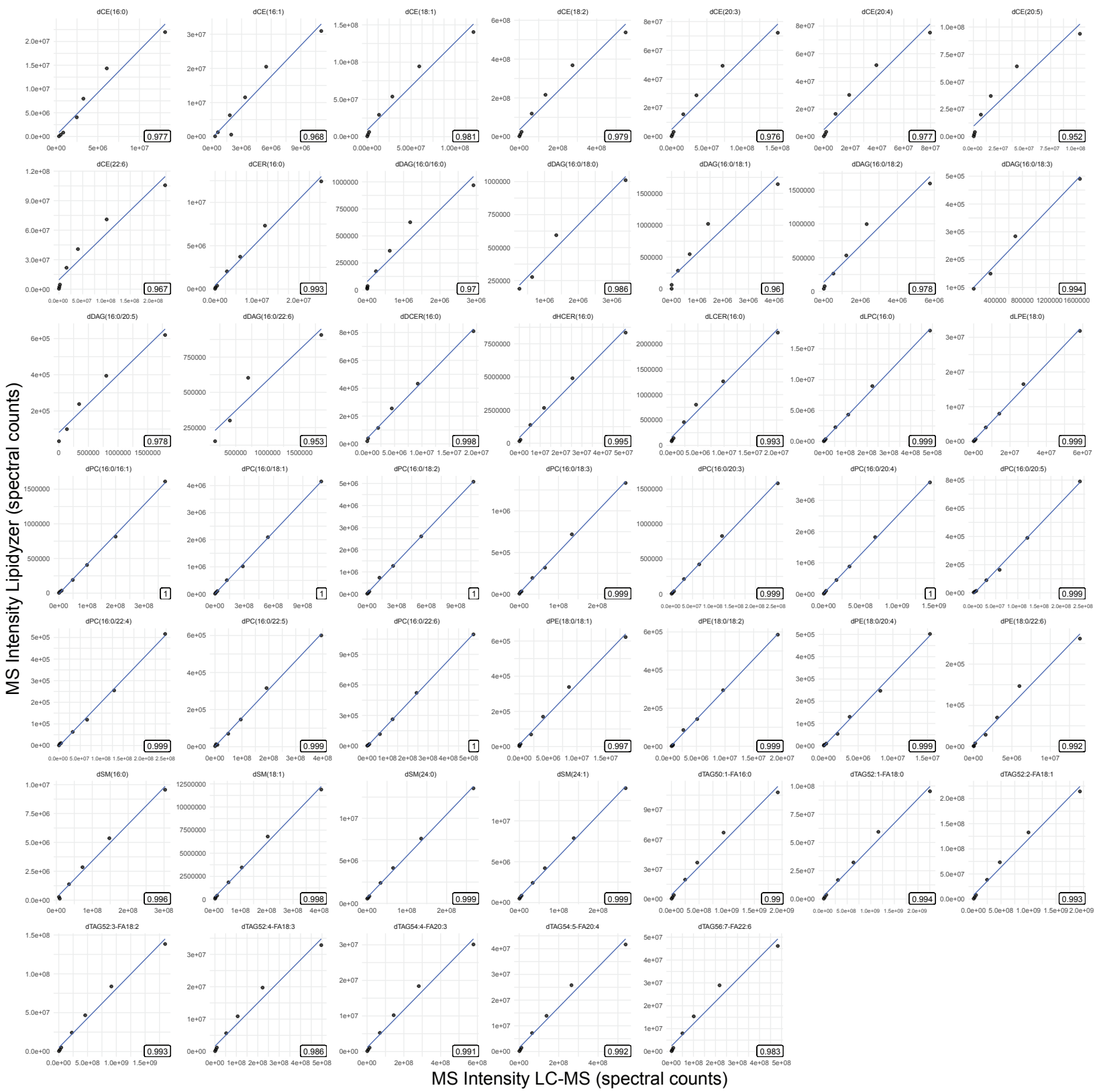

Supplement: Supplementary file 2 — Supplementary Figures [file 41598_2018_35807_MOESM2_ESM.pdf]
